# What are RECIST 1.1 progressions made of? Variability in double-read oncology trials

**DOI:** 10.1007/s00330-025-12234-4

**Published:** 2026-02-09

**Authors:** Hubert Beaumont, Luca Cantini, Kamal S. Saini, Nathalie Faye, Ritu Gill, Antoine Iannessi

**Affiliations:** 1 Median Technologies, Valbonne, France; 2 Fortrea Inc., Durham, NC USA; 3 Addenbrooke’s Hospital, Cambridge University Hospitals NHS Foundation Trust, Cambridge, UK; 4 Columbia University Vagelos College of Physicians and Surgeons University Medical Center, New York, NY USA

**Keywords:** Clinical trial as topic, Observer variation, Solid tumors/diagnosis, Tomography, X-ray computed, Lung neoplasms

## Abstract

**Objective:**

Blinded Independent Central Review (BICR) with double reads and adjudication is crucial in imaging-based clinical trials to ensure quality data. However, discrepancies in double reading can affect trial outcomes, particularly in assessing disease progression in phase 3 oncology studies using RECIST 1.1 criteria. This study examined discordance in the date of progressive disease (DoPD) across RECIST components: target lesion (TL), non-target lesion (nTL), new lesion (NL), exploring its impact on survival curves and whether discrepancies stem from timing differences or true/false PD detection.

**Materials and methods:**

We retrospectively analyzed data from five clinical trials using BICR with double reads plus adjudication, involving 1932 lung cancer patients on immunotherapy or targeted therapy. RECIST components were examined to assess DoPD concordance, discrepancies, adjudicator acceptance, detection timing, and impact on survival curves.

**Results:**

Readers showed a 39.3% discordance rate in DoPD assessments, with agreement on 17.3% of DoPD cases and 43.4% of non-PD cases. In 54.2% of concordant cases, multiple RECIST components contributed to PD. Discordance was primarily caused by NL (41.4%), sum of TL diameter increase (33.3%), nTL (11.8%), or multiple components (13.4%). In 49.2% of discrepant cases, PD was reported late, usually within one treatment cycle (79.8%). 62.5% of disputed PD cases were accepted by adjudication.

**Conclusion:**

Different RECIST components vary in their likelihood of causing discordance and being accepted or refuted by adjudicators. NL detection is key for identifying progression but also the main source of disagreement. Using multiple RECIST components enhances the reliability of PD assessment.

**Key Points:**

***Question*** *How does discordance across RECIST components—especially new lesion detection—influence progression assessment timing and potentially alter survival outcomes in oncology clinical trials?*

***Findings*** *Reader disagreement on progression dates is common, driven mainly by new lesion detection; incorporating multiple RECIST components increases alignment and strengthens PD determination reliability.*

***Clinical relevance*** *Imaging endpoints guide cancer treatment decisions. RECIST 1.1 is the standard, inter-reader variability can undermine PFS accuracy. By evaluating RECIST component reliability, this work aims to improve trial precision, enabling faster, more confident decisions that ultimately benefit patients.*

**Graphical Abstract:**

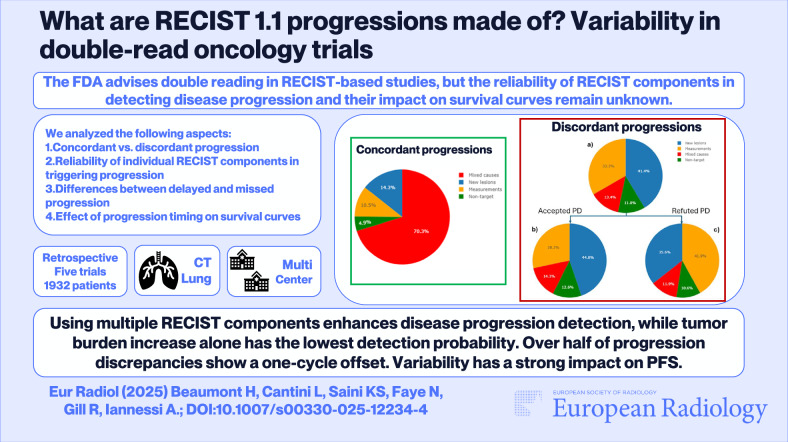

## Introduction

In many fields of medicine [1], double-checking is common practice, bolstering patient safety by minimizing diagnostic errors and offering a more comprehensive view of patients’ conditions. For over two decades, the US Food and Drug Administration (FDA) has promoted [2, 3] Blinded Independent Central Review (BICR) with double reads of images in clinical trials to ensure data blinding, robustness and to reduce bias [4]. This double-checking involves two independent readers evaluating the same set of imaging and resolving any discrepancies through independent adjudication. Currently, two-thirds of cancer-related clinical trials rely on imaging-based endpoints [5], most of them implementing double reads in late-phase studies.

In clinical trials, the evaluation of drug safety and efficacy leans on dedicated endpoints. Even though overall survival (OS) remains the ultimate clinical endpoint, several practical limitations have led to progression-free survival (PFS) becoming an acceptable surrogate of primary clinical endpoints in phase III trials [6]. This involves longitudinal follow-up of patients on the allocated therapy till progressive disease (PD) or recurrence is documented and is referred to as time to progression.

The Response Evaluation Criteria in Solid Tumor (RECIST) 1.1 remains the standard criterion to evaluate drugs in oncology phase III trials since its introduction in 2000 and its revision in 2009 [7]. To declare PD based from baseline, RECIST analyzes three components: (1) The increase of more than 20% of the sum of diameter (SoD) of up to five target lesions (TL) and 5 mm compare to nadir (target lesions being deemed the largest and most reproducible lesions representing the whole spectrum of disease); (2) The unequivocal subjective increase of non-target lesions (nTL), (nTL being defined as all lesions not falling in the TL category); and (3) the appearance of an unequivocal new lesion (NL). The original purpose of RECIST was to standardize readings across the radiology community.

As time went by, several limitations became apparent with the use of RECIST, such as imperfect sensitivity [8], inability to manage lesion-to-lesion heterogeneity [9], and unsuitability for handling specific response patterns with immunotherapies [10].

Another limitation, specific to double-reading settings, is the unavoidable inter-reader disagreements [11] that have been extensively studied and primarily stem from inherent subjectivity in interpreting RECIST images [12].

Consequently, these disagreements need careful monitoring due to their potential negative impact on the statistical power of trials [13]. At the same time, this variability of opinion provides an opportunity to better understand the causes of equivocality of PD per RECIST as an imaging biomarker for oncology studies, together with a more comprehensive view of the disease [14]. Ultimately, inter-reader variability affects the PFS value and its reliability.

One challenge is to enhance the reliability of RECIST by minimizing subjectivity while preserving the benefits of having two independent readers. Our approach consists of analyzing the reliability and role of the respective RECIST components leading to PD [15] to purposely suggest improvement. For example, improvements could target specific RECIST components (as was done previously with lymphadenopathy assessments from V1.0 to V1.1 [16]) or the data flow (such as requiring double-checks for certain RECIST components).

Indeed, we can assume that part of the discrepancies is due to a delay in DoPD detection, because the two readers have different perceptions of the disease or have close perception but different sensitivity. The question is: did either errors or justifiable medical differences have different short/long-term consequences on PFS?

The aim of our study, applying to lung cancer patients only, is to identify which RECIST components are more reliable for detecting disease progression. We conducted separate analyses for major disease locations, distinguishing variability caused by temporal delays in detection from differences driven purely by sensitivity and specificity. Additionally, using simulations and reader variability indices, we assessed the impact of these factors on survival curves.

## Materials and methods

### Data

Our retrospective analysis included annotations from five clinical trials that evaluated immunotherapy or targeted therapy in Non-Small Cell Lung Cancer (NSCLC). The selected trials were conducted between 2017 and 2021 using double reads plus adjudication based on RECIST 1.1 guidelines. All data were fully blinded regarding study sponsor, study protocol number, therapeutic agent, subject demographics, and randomization. Across the five trials, 1932 patients were included, of whom 1728 underwent at least one post-baseline visit, all involving CT scans. In these studies, the interval between visits varied—starting at 6–8 weeks and later extending to 9–12 weeks. The central reads were all performed using the same radiological reading platform (iSee; Median Technologies).

### Read paradigm

Seventeen experienced and trained independent radiologists and 6 adjudicators were involved in these trials.

All radiologists have substantial experience with the RECIST criteria, both on-site and centrally, as well as significant expertise in NSCLC pathology, with at least 10 years of professional experience. The reader pool is selected to be homogeneous.

Two radiologists performed double reviews of each set of images and determined the Radiologic Time Point Response in accordance with the RECIST 1.1 criterion. The same radiologist interpreted all images from each time point for the same patient. In case of discordance, an adjudicator reviewed the response assessments from the two primary readers and accepted one of the reader outcomes (the most correct assessment regarding the progression event). The adjudicator provided a rationale to support his/her decision. Throughout our study, we treated the adjudicator’s decision as the ‘truth,’ while acknowledging that the actual truth may remain unknown.

### Dataflow

The iSee platform allows the reader to record their annotations at each time point: The number, size and location of each TL (not their anatomical location), the number and location of nTL (not their anatomical location) and the detection and location (not their anatomical location) of NL. From the raw annotations, RECIST components are computed, including the change in SoD, the unequivocal progression of nTLs and the unequivocal detection of NL.

In case of discrepancy, the process required the adjudicator to endorse one of the primary readers with justification for the decision, including a semi-standardized text elaborating on the reasons for endorsing a primary reader. Therefore, with the help of the software and the adjudicators’ comments, it is possible to retrospectively assess at which time point the discrepancy occurred and to determine the cause of discordance by comparing readers’ records.

Our analysis method consisted of an automated computation of the RECIST component discrepancies/agreement and, when possible, searching for specific keywords in the comments from the adjudicator. We aimed at detecting discrepancies due to:

**TL assessment:** An obvious difference in measuring TLs.

**nTL assessments:** An unequivocal nTL progression was reported by one reader and not the other.

**NL detection:** An NL was detected by one reader and not the other.

**Mixed detection:** When one reader detected the simultaneous progression of several of the above-mentioned RECIST components, while the other reader did not detect any sign of progression.

### Evaluated aspects

We analyzed the RECIST components according to the declaration of the DoPD:

1. **Concordant DoPD:** We documented the proportion of progression simultaneously reported by the two readers.
2. **Discordant DoPD—adjudicator acceptance:** After being analyzed by adjudicators, we documented the proportion of accepted/refuted progression.
3. **Reliability of RECIST component triggering DoPD:** For each RECIST component, we computed the positive predictive value (PPV) as the ratio of the number of times a RECIST component led to an accepted PD out of the number of times this component was involved. As we hypothesized that reliability can be organ dependent, we further investigated the detection of NL at the main disease locations.
4. **Discordant DoPD—offset:** The analysis evaluates the distribution of offset values (measured in cycles) when two readers declare a DoPD, with one reader identifying it later than the other. Our aim was to determine which RECIST component is most frequently responsible for the initial detection of DoPD.

We assessed two key metrics of the offset distribution:

- The proportion of discrepancies is due to a one-cycle offset.
- Large delay in detection by the second reader, expressed as the 95th percentile of the offset value (in cycles).

We will also assess the potential impact of discrepancies categorized as “reader errors” or “medical justifiable differences” (whether PD is confirmed or unconfirmed).

5) **Single-reader detection (PD versus Non-PD):** We analyzed the distributions of RECIST components when only one reader detected PD, while the other did not at any time point. For this analysis, we aimed to avoid bias from withdrawal or End of Treatment (EoT). Therefore, we included only patients whose disease progression detection and last visit were sufficiently spaced apart. This minimum time gap was defined as a number of cycles greater than or equal to the 95th percentile measured from the offset distribution.
6) **Impact on survival curves**. We simulated survival curves using the key features derived from the primary reads and adjudications analyzed in this study: Discordance Rate (DR), distribution of offsets, and single-reader detection rates. The simulation was based on a public dataset from a typical clinical trial. To evaluate the impact, we varied the DR, adjusted the distribution of offsets, and modified the proportion of single-reader detections to reflect different scenarios.

### Statistics

Statistical analysis was performed using R freeware and considering 5% type I error. Proportions were presented with 95% confidence intervals (CI) estimated using the Clopper–Pearson model.

We computed the discordance rate, defined as the number of discrepant cases divided by the number of cases.

We used unpaired two-sample Wilcoxon test for two group comparisons, and Marascuilo test for the comparison of multiple proportions.

The publicly available “lung” dataset from the “Survival” R package served as the basis for computing our reference survival curve. Using this reference, we simulated survival curves corresponding to a range of conservative readings, each characterized by varying Discordance Rate (DR) values, single-detection proportions, and offset distributions. We define conservative reading as a reader’s tendency to declare PD if and only if they are strongly convinced, with high probability, that PD is indeed occurring. This bias may lead the conservative reader to wait for an additional confirmation cycle or not to declare progress at all.

## Results

**Fig. 1:**
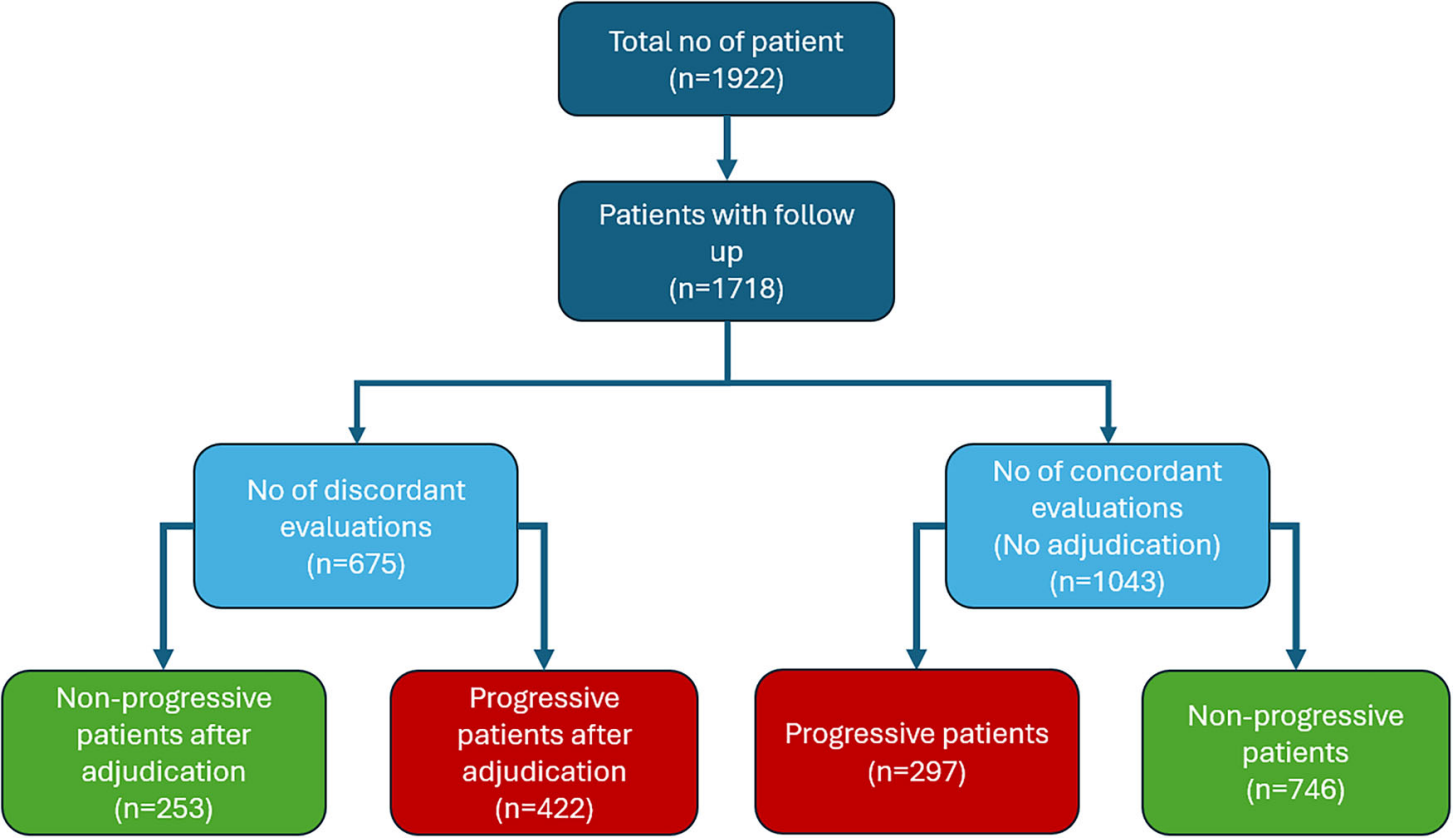
Concordant/discordant DoPD declaration and progressive/non-progressive patients. Pooling all trials, 1922 patients were included, of whom 1718 underwent at least one visit after baseline. The discordance rate of DoPD was 39.3% (675 patients), and at the end of the trials, 41.8% of the patients were deemed progressive (*N* = 719). A total of 43.4% of patients were reported as non-progressive, and 17.3% as progressive, with full agreement among readers

Figure 1 provides an overview of the dataset, split into four patient groups: those for whom both readers agreed on PD or non-PD, and those classified as progressive or non-PD only after adjudication.

Readers agreed on PD in 17.3% (297/1718) of patients and on non-PD in 43.4% (746/1718). In discordant cases, adjudication classified 24.6% (422/1718) as PD and 14.7% (253/1718) as non-PD.

### RECIST components in concordant PD declarations

**Fig. 2:**
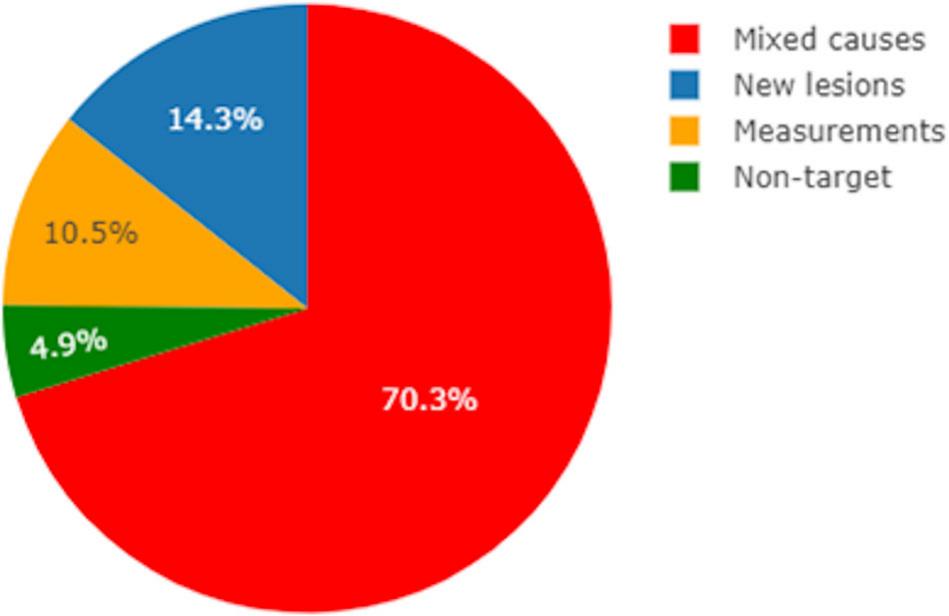
RECIST components involved in concordant reviews. Readers largely agreed on DoPD due to mixed causes. When considering a single RECIST component, the new lesion or target lesion categories were the most influential. DoPD, date of progressive disease

Figure 2 breaks down concordant PD declarations by the RECIST components used by both readers. Agreements stemmed from multiple causes in 70.3% (95% CI: 64.6–75.5), from NL only in 14.3% (95% CI: 10.5–18.9), from SoD increase only in 10.5% (95% CI: 7.2–14.6), and from progressive nTL only in 4.9% (95% CI: 2.7–8.1).

Among concordances attributed to multiple causes (70.3%), 54.2% occurred when at least one reader identified PD based on several RECIST components, while 16.1% occurred when both readers agreed on PD but relied on different single components.

### RECIST components in discordant DoPD declarations

**Fig. 3:**
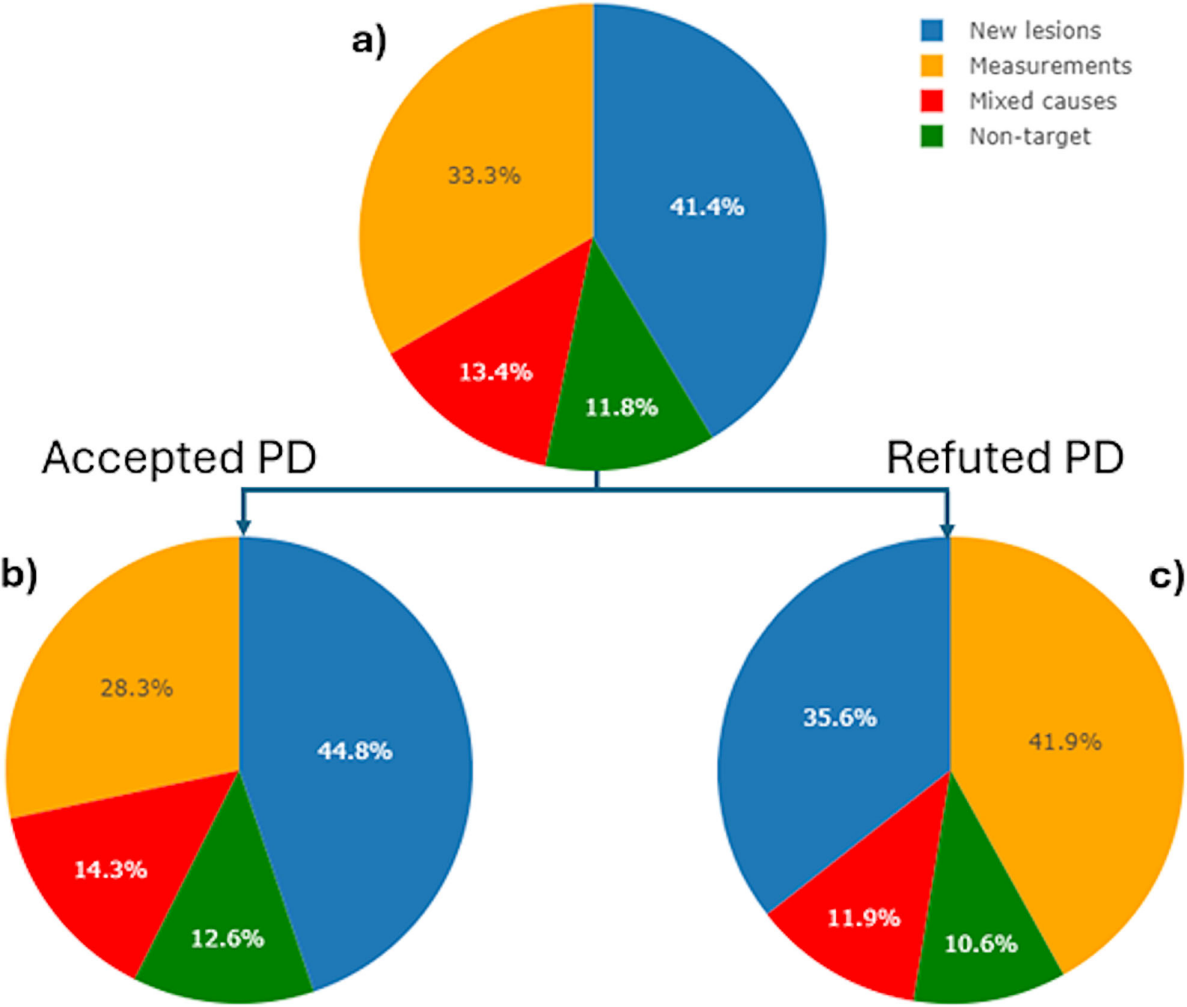
RECIST components involved in discordant reviews. **a** Discordances were attributed to NL, SoD measurements and non-target evaluations only in respectively 41.4% (95% CI: 37.6, 45.3), 33.3% (95% CI: 29.7, 37.1), and 11.8% (95% CI: 9.4, 14.6) of the cases. Multiple RECIST components were involved in 13.4% (95% CI: 10.8, 16.3) of the cases. Almost two-thirds of discrepant PD were accepted by the adjudicators (62.5%). **b** As confirmed by adjudicators, 44.8% (95% CI: 39.9, 49.8) of PD were confirmed to result from NL occurrence, 28.3% (95% CI: 24.0, 33.0) from a significant increase of SoD, and 12.6% (95% CI: 9.5, 16.2) from unequivocal non-target progression only. Accepted PD due to multiple causes represented 14.3% (95% CI: 11.0, 18.1) of the cases. **c** When adjudicators refuted PD, the RECIST component involved was SoD in 41.9% (95% CI: 35.6, 48.5), NL occurrence in 35.6% (95% CI: 29.5, 42.1), and unequivocal non-target progression in 10.6% (95% CI: 6.9, 15.2). Refuted PD due to multiple components represented 11.9% (95% CI: 8.0, 16.7) of the cases. NL, New Lesion; PD, Progressive disease; SoD, Sum of tumor diameter

Discordance in DoPD declaration occurred in 39.1% (675/1718) of evaluations. Figure 3a shows the RECIST components involved in these discrepancies, Fig. 3b the proportions accepted by adjudicators, and Fig. 3c the proportions refuted. Overall, adjudicators accepted 62.5% (95% CI: 58.9–66.2) of discordant DoPD calls.

The largest share of refuted diagnoses involved progressive SoD, often due to measurement errors. According to adjudicators’ notes, 15% of these refutations were linked to atelectasis being mistakenly included in tumor measurements.

### Confidence in RECIST components—focus on NL detection

**Fig. 4:**
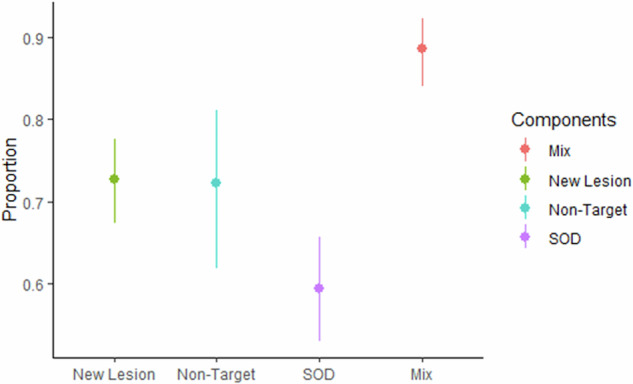
Positive predictive value associated with the RECIST components. Increase in SoD is less likely to trigger a PD (0.59 (95% CI: 0.53, 0.66)), while reporting several progressive RECIST components is more likely to report a definitive progressive patient (0.89 (95% CI: 0.84, 0.92)). SoD, sum of diameters of target lesions. PD, Progressive disease

Figure 4 summarizes the PPV associated with each RECIST component.

Focusing on progressions based solely on NL: at trial end, 53.8% (95% CI: 50.1–57.5) of adjudicator-accepted PD were attributed to NLs. Acceptance rates for NL detection differed significantly across main disease sites (Table 1, Marascuilo test, q < 0.05).

**Table 1:** The rate of acceptance when NL detection is the only trigger for DoPD discrepancies

	Lymph nodes	Lung	Liver	Bone	Brain
Acceptance rate (%)	88.4 (77.4, 95.2)	40.6 (28.9, 53.1)	50.0 (11.8, 88.2)	86.7 (59.5, 98.3)	71.4 (29.0, 96.3)

*DoPD* date of progressive disease

When both readers reported a single NL, they agreed on the same organ in 83.4% (95% CI: 70.7–92.1) of cases, most often in the brain, lung, or lymph nodes.

### Timing of progressions: delay versus false detection or omission?

**Fig. 5:**
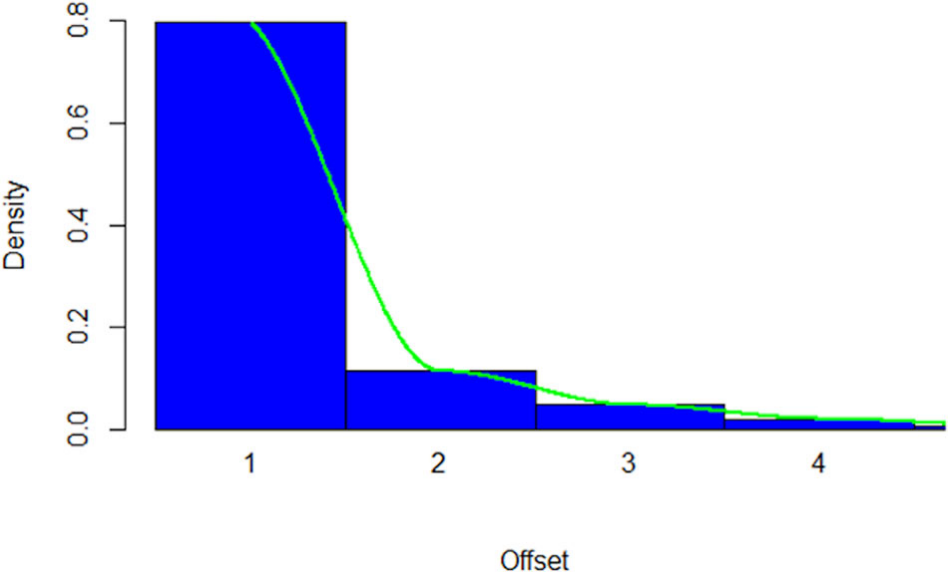
Distribution of offset values in declaring PD. Histogram of offset values (blue), showing 79.8% of offsets occurring at one cycle and 2.1% at four cycles. The density curve is superimposed in green for comparison. PD, Progressive disease

When discrepancies arose at a given time point, 49.2% (95% CI: 45.3–53.0) were due to one reader reporting PD later. Figure 5 shows the distribution of these delays. Initial PD calls were triggered by NL in 46.4% (95% CI: 40.9–51.9) and by SoD increase in 32.5% (95% CI: 27.5–37.8). Differences in TL selection at baseline accounted for 18.7% (95% CI: 14.6–23.3) of delayed detections.

A per-component summary is shown in Table 2. Using the 95th percentile threshold, we defined a single-reader detection as PD identified by only one reader at least four cycles before withdrawal or EoT. Such detections were considered either false positives or missed true PDs.

**Table 2:** Distribution of offset in declaring PD per RECIST components

	% of detection with one-cycle offset	95th perc. offsets (cycles)
Mix of components	79.8 (95% CI: 75.1, 84.0)	< C3
New lesion	54.5 (95% CI: 46.3, 62.6)	< C4
SOD	54.6 (95% CI: 44.8, 64.2)	< C4
Non-target	74.5 (95% CI: 60.4, 85.7)	< C3

In the subset of discrepant PD evaluations, the left column shows the proportion of PD detected with a one-cycle delay, while the rightmost column indicates the number of cycles by which the two readers differed in detecting 95% of PD cases. When not distinguishing between RECIST components (Undifferentiated), 79.8% of PD detections occurred with a one-cycle delay. Per RECIST components, offset is minimal when one reader detects unequivocal progression of the non-target lesion

*PD* progressive disease, *SoD* sum of tumor diameter

Adjudicators refuted 55.6% (95% CI: 41.4–69.1) of single-reader detections based on a single component, most often false NL (40.0%, 95% CI: 22.6–59.4) or false SoD increase (23.4%, 95% CI: 9.9–42.3). When adjudicators upheld single-reader detections, 70.8% (95% CI: 48.9–87.4) were true NL and 8.4% (95% CI: 1.0–27.0) true SoD increase.

### Impact of RECIST reliability on survival curve

**Fig. 6:**
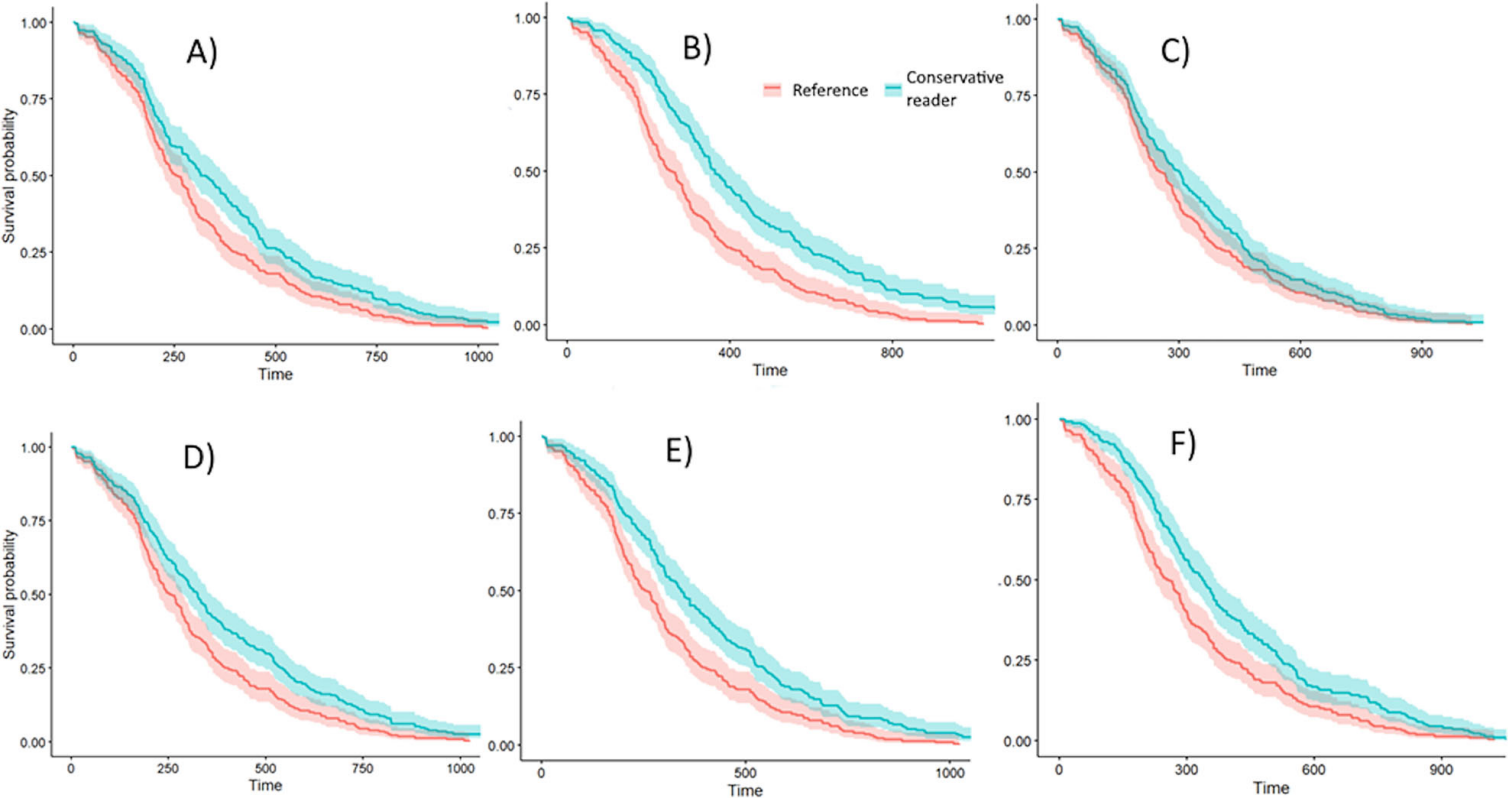
Sway of variabilities upon survival curves. From top left to bottom right: **A** DR: 40%, Single detection: 50%, One-cycle offset: 80%; **B** DR: 70%, Single detection: 50%, One-cycle offset: 80%; **C** DR: 40%, Single detection: 20%, One-cycle offset: 80%; **D** DR: 40%, Single detection:70%, One-cycle offset: 80%; **E** DR: 40%, Single detection: 50%, Offset: Uniformly distributed. **F** DR: 60%, Single detection: 50%, Offset: One-cycle offset: 80%. DR, discordance rate

We evaluated the impact of inter-reader variability on survival curves, considering detection rate (DR), delays, and differences in sensitivity/specificity. With a baseline DR of ~40%, a single-reader DR of 50%, and an offset distribution (Fig. 5) showing ~80% at one cycle, Fig. 6 illustrates simulated survival curves for conservative readers (delayed detection).

Figure 6C shows that reducing single-reader detections (i.e., improving PD reliability) narrowed the gap between survival curves. Specifically, PFS from Fig. 6D, F were nearly identical at 362 (95% CI: 313–393) and 366 days (95% CI: 350–426). By contrast, increasing DR (Fig. 6B) or applying a uniform offset distribution (Fig. 6E) widened the survival curve gap shortly after baseline.

## Discussion

### Summary of key findings

The discordance rate for DoPD was 39.3%. About 60% of these discrepancies were ultimately confirmed as PD by adjudicators. Among confirmed cases, nearly half (44.8%) involved new lesions (NLs), whereas the main reason for refuting PD was inaccurate increases in SoD (41.9%).

For individual readers, progression calls based on multiple RECIST components provided the most reliable signal (PPV = 0.89, 95% CI: 0.84–0.92). In contrast, detections based solely on SoD increase were least reliable (PPV = 0.59, 95% CI: 0.53–0.66).

At trial completion, most PD diagnoses were driven by the detection of NLs (53.8%). Confirmation rates varied by site: lung NLs had the lowest confirmation rate (40.6%), while nodal NLs had the highest (88.4%).

Reader disagreement often reflected timing differences. In about half the cases, the second reader reported PD after a delay, 80% of these delays being one cycle. Offset distributions varied across RECIST components: NL detections were associated with the longest delays, while nTL or mixed-component progressions showed minimal offsets.

Adjudicators rejected 55.6% of single-reader PD detections, with 40% of rejections due to disputed NLs. Conversely, when single-reader calls were upheld, 70.8% involved confirmed NLs.

Finally, simulations demonstrated that DR, detection offsets, and the frequency of single-reader detections significantly affect survival curves and thereby impact clinical trial endpoints.

### Comparison with previous studies

The discordance rate of 39.3% was in the range of the 38% (37%, 40%) provided by Ford et al [11] for lung cancer.

The proportion of patients with NLs involved in the radiologic progression can be assumed disease-dependent, as for clear cell renal cell carcinoma (26.6%) [15] or colorectal cancer (50.6%) [17]. For NSCLC, we found that NLs were the main cause of progression (53.8%). This is not surprising, particularly in lung cancer trial datasets, where inflammatory changes in the lung parenchyma are common. Experienced readers may therefore wait for an additional time point before confirming PD. Inflammatory or infectious lesions that mimic new malignancies are also more frequent under certain treatments, such as radiotherapy and immunotherapy. The interpretation of new lesions is further complicated in the case of sclerotic bone metastases, which may represent treatment response rather than true progression on CT scans.

Close to our study (41.4%), Beaumont et al [18] found that the majority of discrepancies were due to the detection of NLs (53.7%), nTL progressions comprised about 10% of all disagreements; however, Beaumont et al considered local-central variability.

In their simulation study, Bucho et al [19] identified TL selection as a major source of variability. However, a key limitation of their work was the exclusion of the NL impact. Our findings did not support their conclusion, as only 12.6% of disagreements in our dataset were attributable to baseline tumor selection. Moreover, another study [20] showed that, even though tumor selection was a significant risk factor, the effect was not strong enough to be a predictor, therefore not having a strong impact on PD detection.

We found that 13.4% of discrepancies were due to the simultaneous increase of different RECIST components by one of the readers; a comparable proportion of 16.6% was found in a previous study [18]. However, we also found that when both reviewers agreed on PD, 54.2% of evaluations were triggered by at least one reader using a combination of RECIST components. This is expected, as multiple concurrent indicators increase the likelihood of true PD. The relatively large share of refuted PD calls (37.5%) based on multiple components can be explained statistically by the non-zero probability of several independent events occurring simultaneously. Overall, our analysis shows that evaluations based on a mix of RECIST components are the most reliable, with the highest PPV (0.89).

We established that nearly half of the discordances are due to delays (49.2%), with 46.4% of these delays involving NL detections and 54.5% of these delays being one cycle in length. This one-cycle delay could represent the time required to confirm an ‘equivocal new lesion’ according to the RECIST guidelines [7]: “For equivocal findings of progression … until the next scheduled assessment”. This reasoning applies to all RECIST components and underscores the role of confirmation in reducing variability across evaluations. It is especially relevant in the context of emerging progression patterns, such as pseudoprogression with immunotherapy [21]. However, we found that 80% of offsets lasted only one cycle. This is reassuring, as it limits the impact of reader variability at the study level and reflects the overall quality of the reads. Since PFS and Duration of Response are closely tied to PD, larger discrepancies could have posed significant issues. Importantly, the high proportion of one-cycle offsets may also serve as a useful metric for monitoring trial quality.

When not due to delayed detection, single-reader detections can be interpreted as an issue of reader sensitivity or specificity. Iannessi et al [22] documented reasons to call falsely for PD due to intercurrent diseases, artefacts or findings incorrectly considered as new. This topic is also indirectly addressed in the literature by Gong et al [23] who propose AI-derived solution for detecting new liver tumors and by Iannessi et al [24] and Bucho et al [19] who documented the impact of lesion selection at baseline upon sensitivity or by Beaumont et al [18] who concluded that the assessment of new nodal lesions was the major sensitivity/specificity issue in trials whereas our study showed a confirmation rate of 88.4%.

The repercussion of the variability of evaluations upon survival curves has been reported in local-BICR settings [25]. Consistent with this, our simulations confirmed the significant influence of the factors we analyzed. Notably, we found that the effect on PFS was similar when increasing DR from 40 to 60% or when reducing the proportion of single-reader detections from 70 to 50%. These findings highlight that, in the context of clinical trials, understanding the composition of DR is more informative for trial monitoring than focusing solely on its overall value.

### Limitations

First, our analysis of the prognostic power associated with RECIST 1.1 was based on classifying disagreements between adjudicator-accepted PD and non-accepted PD. However, we recognize that the adjudicator’s opinion can be significantly biased and therefore is not an indisputable reference, and our analysis would have been more relevant if it had been based on a clinical index such as OS. The conclusions might have been different in that case.

Second, the distribution of offset in the detection of PD did not consider the difference in the visit schedule across trials. Our purpose was to investigate the difference in reader sensitivity at detecting during sequential visits; therefore, we believed that the difference in visit scheduling was not detrimental to our analysis.

Lastly, the trials we included in our study were blinded; therefore, we were unable to check the possible impact of the treatment of interest against the standard of care. Even if we included trials that were as similar as possible, we cannot rule out the possibility of different variabilities between the two arms in each trial.

### Future directions

Building on our work, several potential improvements can be identified. Automatic detection and characterization of new lesions (NLs) are particularly important in challenging areas such as the lungs and liver, where reader performance tends to be suboptimal. Computer-aided diagnosis (CAD) systems could help reduce discrepancies by mitigating delays from one-cycle confirmations and decreasing reliance on single-reader detections. Current data indicate that 50.8% of discrepancies arise from single-reader detections, 55.6% of which are refuted, with 40% involving NLs. An ideal CAD system could potentially prevent 10% of these discrepancies, including 4.4% false positives and 6.3% false negatives.

Specifically for lung lesions, advanced Lung CAD systems might reduce the overall discordance rate by 4%. However, although Lung CAD technologies perform well in screening settings [26], their effectiveness in clinical trial data remains uncertain due to differences between datasets. CAD performance in other areas, such as the liver, also remains inadequate [27], leaving 44.8% of NL discrepancies unresolved.

In the short term, improving reader training offers a practical solution [28]. Prioritizing analysis of false NLs, such as misidentified atelectasis, and refining methods for confirming equivocal progression are key steps. Readers should also be encouraged to consider multiple RECIST components when triggering a PD to enhance detection accuracy.

Because PD detection based solely on SoD increase has a low PPV, accurate assessment is critical. This evaluation can be influenced by several factors, including subjective baseline labeling of TL versus nTL [19], as well as variability in measurements [29]. Increasing concern has been raised regarding the summation of tumor diameters, which often stems from variable selections of diseases at baseline [9, 30].

Beyond the issue of variability, this raises the question: what truly defines PD? Consider the following examples of debatable stable responses: (1) a single TL enlarges significantly (> 50%) but is summed with multiple indolent TLs; (2) multiple TLs each grow by less than 20%; (3) one TL enlarges (> 20%) while another shrinks (< 20%), both summed with other indolent TLs.

Our simulations qualitatively highlighted how various aspects of variability can affect survival curves. However, it is now necessary to quantify these effects more precisely, particularly in relation to study endpoints such as PFS. Such quantification will help guide improvements aimed at enhancing the reliability of these endpoints.

Immunotherapy trials differ fundamentally from the cytotoxic-therapy era in which RECIST was developed. Immune infiltration can make tumors appear larger or produce NL, creating apparent progression. Mixed responses are also common, with some lesions shrinking while others grow, and true tumor shrinkage may not appear until weeks or months later. NTL lesions may offer early signals of benefit or failure. As a result, every component of RECIST comes under pressure when applied to immunotherapy, underscoring the need to develop new response criteria tailored for double readings [8].

## Conclusions

The individual RECIST components show differing propensities for reader discordance and for confirmation or refutation at adjudication. Among these, the identification of new lesions is pivotal for determining progressive disease (PD) but also constitutes the main source of disagreement between readers. When PD is defined solely by the appearance of a new lesion, the detection of an extrapulmonary lesion serves as a more reliable indicator of progression. To improve the robustness and reproducibility of tumor response assessment, reporting PD supported by more than one RECIST component is strongly encouraged.

## Abbreviations

BICR: Blinded Independent Central Review
CAD: Computer-aided diagnosis
DoPD: Date of progressive disease
DR: Discordance rate
EoT: End of treatment
FDA: Food and Drug Administration
NL: New lesion
NSCLC: Non-small cell lung cancer
nTL: Non-target lesions
OS: Overall survival
PD: Progressive disease
PFS: Progression-free survival
PPV: Positive predictive value
RECIST: Response Evaluation Criteria in Solid Tumor
SoD: Sum of tumor diameter
TL: Target lesion
